# Editorial: Insights in autoimmune and autoinflammatory disorders: 2021

**DOI:** 10.3389/fimmu.2022.1092469

**Published:** 2022-12-14

**Authors:** Raphaela Goldbach-Mansky, Betty Diamond

**Affiliations:** ^1^ Translational Autoinflammatory Diseases Section (TADS), Laboratory of Clinical Immunology and Microbiology (LCIM), National Institute of Allergy and Infectious Diseases, National Institutes of Health (NIH), Bethesda, MD, United States; ^2^ Institute of Molecular Medicine, Feinstein Institutes for Medical Research, New York, NY, United States

**Keywords:** autoimmunity, autoinflammation, SLE, RA, risk factors, viral pathogenesis, biomarkers

The Research Topic: *Insights in Autoimmune and Autoinflammatory Disorders: 2021* was implemented to highlight latest advancements in research across the field of Immunology in Autoimmunity and Autoinflammation by discussing recent advances, current challenges, and future perspectives. Under the Research Topic 13 articles were published, 10 contributions highlight topics relevant to autoimmune diseases and 3 to autoinflammation.

In two original research papers two groups set out to identify microbial or viral risk factors that predispose to the development of autoimmunity.

Yin et al. mined microarray datasets from disease-specific target tissues including the pancreas, thyroid, and intestine from individuals with Type 1 Diabetes (T1D), Hashimoto thyroiditis (HT), and celiac disease (CD), as well as matched controls. They discovered viral signatures of common viral infections including influenza A, human T-lymphotropic virus type 1, and herpes simplex that were shared in target tissues of the three autoimmune diseases studied thus pointing to common environmental factors as drivers of autoimmune diseases. Lin et al. assessed a potential role of gut microbiota in driving autoimmune diseases in a retrospective population-based cohort study analyzing data from a Taiwanese Insurance Research Database of over 290,000 patients treated with PPIs in the period between 2002 and 2015. The role of PPIs in modifying gut microbiota has previously been described (1). The authors show that compared to the non-PPI group, the adjusted hazard ratios (aHR) were higher for several organ specific autoimmune diseases including Grave’s disease, Hashimoto thyroiditis, autoimmune hemolytic anemia, immune thrombocytopenic purpura, Henoch-Schoenlein purpura and Myasthenia gravis and also for systemic autoimmune diseases including ankylosing spondylitis, rheumatoid arthritis, primary Sjogren syndrome, systemic lupus erythematosus, systemic vasculitis, psoriasis, systemic scleroderma and inflammatory myopathies.

Two papers, an original research paper by Yang et al. and a mini review by Chen et al. assess the role of transfer RNA (tRNA)-derived small noncoding RNA (tsRNA), an emerging class of small non-coding RNAs, and the role of ferroptosis in patients with systemic lupus erythematosus (SLE) respectively. Yang et al. assessed the diagnostic value of a potential biomarker, tRF-His-GTG-1, a candidate tsRNA that best differentiated between the SLE and control groups. The tsRNA was first assessed in a training set of 57 SLE patients with and without lupus nephritis and then in a validation set of 52 SLE patients without Lupus nephritis, 83 SLE patients with lupus nephritis and 86 healthy controls; tRF-His-GTG-1 was significantly elevated in serum exosomes from SLE patients compared to healthy controls and its elevation in serum distinguished SLE with lupus nephritis from SLE without lupus nephritis with and AUC of 0.81 (95% CI 0.73-0.88) with high specificity, however, the sensitivity was lower, 66.27%. Pathway analysis predicted that the tsRNA can target signaling pathways including MAPK signaling and RIG-I signaling and EBV infection. Chen et al. review data on the role of ferroptosis, a novel non-apoptotic regulated form of cell death, in SLE.

Two reviews assess mechanisms that lead to fibroblast proliferation and inflammation in rheumatoid arthritis (RA). Jiang et al. review the pathogenic role of secreted frizzled-related protein 1 (SFRP1), a member of the secretory glycoprotein SFRP family, that are thought to “antagonize” the Wnt signaling pathway by interfering es with Wnt signaling transduction. Their role in determining cell fate by regulating cell proliferation, differentiation, apoptosis, and pyroptosis has been previously reported (2) SFRP1 is widely expressed in human cells, including fibroblast-like synoviocytes (FLS) of rheumatoid arthritis (RA) and in osteoarthritis (3). The authors summarized data on mechanisms of SFRP1 regulation of RA-FLS pyroptosis through Wnt/β-catenin and Notch signaling pathways and summarize data on the epigenetic regulation of SFRP1 in RA-FLS. They conclude proposing that Wnt/β-catenin and Notch signaling pathways may collaborate in NLRP3-mediated cell pyroptosis and suggest a role of SFRP1 in hypermethylation of synovial tissue from knee joints in patients with RA and OA. The authors further suggest a potential role of inhibition of hypermethylation in the treatment of RA. Zhao et al. assess the role of G-Protein-Coupled Receptors (GPCR) that includes chemokine receptors, melanocortin receptors, lipid metabolism-related receptors, adenosine receptors, and other inflammation-related receptors, on the pathogenesis of RA, in regulating inflammation, lipid metabolism, angiogenesis, and bone destruction. This review provides comprehensive tables on GPCRs and their expression in immune cells synovium and synovial fibroblasts and discusses possible factors that elucidate the failure of clinical trials blocking cytokines in RA. The authors suggest that the widespread expression of chemokine receptors in a variety of cells may imply that a portion of chemokine receptors may be necessary for homeostatic processes, and further point to the fact that the expression of GPCRs at different disease stages of RA may have diverse functional roles.

Three papers address various mechanisms that can modify tissue specific autoimmune effects, including the role of double negative T regulatory cells in regulating tolerance in the female reproductive environment, the role of NETs in IgA vasculitis and an original paper probing the relationship of C3 levels with disease outcomes in patients with glomerular basement membrane (GBM) disease. Bafor et al. review the emerging role of double negative T regulatory cells (DNTregs), (TCRαβ+/γδ+CD3+CD4–CD8–) on regulating immune tolerance in and female reproductive function. The breakdown of immune tolerance leads to ovulation dysfunction, implantation failure, and pregnancy loss. The authors also discuss mechanisms by which DNTregs provide immune tolerance and maintain and restore the balance in the reproductive microenvironment of female fertility. Chen et al. review the pathomechanisms of IgA activated neutrophils and their release of NETs into tissues and the peripheral blood. The authors review the role of NETs in Immunoglobulin A vasculitis (IgAV) in children. They summarize data suggesting that IgA can induce neutrophils to release NETs *via* Fcα receptor I (FcaRI) and that FcaRI is elevated in children with active IgAV. The authors further suggest that NETs may serve as potential biomarkers to assess disease activity in IgAV. Zhu et al. conducted a retrospective study of 94 anti- glomerular basement membrane (GBM) disease who were seen in the National Clinical Research Center of Kidney Diseases, Jinling Hospital (China) and found that kidney outcomes of anti-GBM disease in the low C3 group were poorer than those in the normal C3 group. Jiang et al. report a patient presenting with coexisting of autoimmune polyglandular syndrome (APS) Type 3 and Gonadotropin-Releasing Hormone Deficiency who presented with secondary amenorrhea.

And finally, 3 original research papers focus on the pathogenesis of three autoinflammatory diseases. The first paper reports a patient with a novel *de novo JAK1* mutation causing a keratinization disorder presenting with hepatitis and autism that was examined in a murine model, a second paper reports of a screening method for candidate drugs for Behcet’s disease, candidates were evaluated in a murine experimental autoimmune uveitis (EAU) model, and the third paper describes a novel disease-causing *NLRP3* variant that presents as autosomal dominant hearing loss.

Takeichi et al. report a patient with a novel, *de novo JAK1* mutation, p.H596D, who presents with generalized papular skin rashes consistent with a keratinization disorder and with early-onset liver dysfunction and autism. Using CRISPR-Cas9 gene targeting, the authors generated mice with the identical *JAK1* mutation and describe hyperactivation of tyrosine kinases and NF-κB signaling pathways. To investigate the role of the mutation in neurodevelopment, the authors observed a strong correlation between genes downregulated in the Jak1^H595D/+;I596I/+;Y597Y/+^ mice and those downregulated in the brain of model mice with 22q11.2 deletion syndrome that showed cognitive and behavioral deficits, and are used to model autism spectrum disorders. Xia et al. used an in silico approach to identify potential drugs for the treatment of Behcet’s disease that they validated in a mouse model of experimental autoimmune uveitis (EAU) (B10.RIII mice developed EAU after subcutaneous injection of 200 µl of emulsifier per mouse). The biological functions and pathways of the target genes were analyzed in detail by Gene Ontology (GO) and Kyoto Encyclopedia of Genes and Genomes (KEGG) analyses and gene drug interactions were identified from the Drug Gene Interaction Database (DGIdb). Of drugs identified to interact with the 3 top hub genes in Behcet’s, rabenprozole and celastrol reduced anterior chamber inflammation in retinal inflammation in EAU mice. Lastly, Oziebloet al. screened 110 autosomal dominant hearing loss (HL) families with a custom panel of 237 HL genes and identified one family carrying a novel *NLRP3* mutation, p.S624R that led to HL in 9 patients in a pedigree of 4 generations. Functional studies identified the novel variant as gain-of-function mutation, leading to increased activity of caspase-1 and subsequent oversecretion of the proinflammatory interleukin-1β. Similar to patients with previously reported mutations causing DFNA34, the identified patients did not present with features of systemic inflammation and did not meet the diagnostic criteria for cryopyrin associated autoinflammatory diseases (CAPS) including MWS, NOMID, or FCAS.

## Author contributions

Both authors contributed to this editorial RM drafted the editorial and BD approved the final version.
